# Acute and Subchronic Toxicity Studies on the Aqueous Extract of the Plant Mixture (*Bidens pilosa* and *Cymbopogon citratus* Aerial Parts) in Rat Model

**DOI:** 10.1155/2022/1998433

**Published:** 2022-12-02

**Authors:** Yannick Carlos Tcheutchoua, Danielle Claude Bilanda, Yolande Sandrine Mengue Ngadena, Paul Désiré Djomeni Dzeufiet, Pascal Emmanuel Owona, Ronald Bidingha á Goufani, Rodrigue Ngapout Fifen, Lohik Mbolang Nguegan, Michel Noubom, Théophile Dimo, Pierre Kamtchouing

**Affiliations:** ^1^Department of Animal Biology and Physiology, Department of Animal Biology, Faculty of Science, University of Yaoundé I, Yaoundé, Cameroon; ^2^Department of Psychology, Faculty of Arts, Letters and Social Science, University of Yaoundé I, Yaoundé, Cameroon; ^3^Department of Biological Sciences, Faculty of Medicine, University of Dschang, Dschang, Cameroon

## Abstract

*Bidens pilosa (B. pilosa)* and *Cymbopogon citratus (C. citratus)* are plants used individually or in combination in the traditional treatment of several ailments such as cardiovascular disorders. In order to valorise their traditional use, a toxicological study was conducted on the aqueous extract of the mixture of aerial parts of *B. pilosa* and *C. citratus*. The acute and subchronic toxicity studies were conducted according to the OECD 425 and 407 guidelines. Regarding the acute study, the aqueous extract of the mixture of *B. pilosa* and *C. citratus* 50 : 50 (2000 and 5000 mg/kg) was administered once to rats of both sexes. In the subchronic study, the aqueous extract of the mixture of *B. pilosa* and *C. citratus* (200, 400 and 800 mg/kg) was administered once daily to rats for 28 days. The aqueous extract of the mixture of *B. pilosa* and *C. citratus* (2000 and 5000 mg/kg) did not cause death and did not induce any apparent sign of toxicity during the 14 days of observation. The DL_50_ of the extract is therefore greater than 5000 mg/kg. Taken daily for 28 days, the extract had no significant effect on selected parameters (creatinine, AST, ALT, urea, and uric acid) of renal and hepatic function, as well as on the number of some blood cells. However, the aqueous extract of the mixture of *B. pilosa* and *C. citratus* (200 and 400 mg/kg) caused a significant (*p*  <  0.05; *p*  <  0.001, respectively) decrease in creatinine levels in male rats as compared to normal control animals. In females, the aqueous extract of the mixture of *B. pilosa* and *C. citratus* (200 and 400 mg/kg) resulted in a significant (*p*  <  0.05) increase in total cholesterol levels as compared to normal control animals. The study showed that the aqueous extract of the mixture of *B. pilosa* and *C. citratus* has a low toxicity and does not cause any injury to the liver, kidney, lungs, or spleen.

## 1. Introduction

The variety of plant species in the Cameroonian flora is very interesting. Indeed, these plants are important for the balance of ecosystems and are used for the survival of the population and their daily activities, by providing some natural resources. Their contribution for food security and primary health care is of high importance for the developing countries' population [1]. Today, the practice of herbal medicine combined with conventional medicine has attracted more attention and is increasingly accepted worldwide [2]. Though generic medicines are available, many of them remain financially inaccessible to the economically disadvantaged population. This leads people to turn to the more available and effective bioactive molecules [3]. However, there is no direct link between the traditional use of a plant for therapeutic purposes and its safety [4]. While many studies have demonstrated the effectiveness of many plants, investigations about their safety are seriously lacking. The main goal of the toxicity study is to regulate the use of the substance studied, and this passed through several pharmacological tests. These tests are arranged in some conventional experimental protocols to check the administration route as well as potential injuries and mortality.

Many plants have already proven their effects on various diseases. For example, *Bidens pilosa (B. pilosa)* and *Cymbopogon citratus (C. citratus)* are medicinal plants belonging to the *Asteraceae* and *Poaceae* families, respectively. These plants commonly called *black Jack* and *fever grass* have been traditionally used to manage a number of diseases. *B. pilosa* is used for influenza, malaria, ear infections, hepatitis, haemorrhoids, and heart problems [5]. *C. citratus* is used to treat gastrointestinal pain, herpes, fever, headaches, and heart problems [6]. Previous studies have confirmed the traditional uses of these plants [7–11] and assessed the toxicity of each plant [6, 12–14]. Several scientific studies have already been carried out on plant mixtures [15–18]. These two plants in mixture are also used to treat cardiovascular disorders (hypertension) in the central region of Cameroon but no data on the toxicity of the mixture are available. This gap prompted the implementation of the present work, which intends to study the toxicity of the aqueous extract of the mixture of the aerial parts of *B. pilosa* and *C. citratus* in rats. The main purpose has been to highlight the potential dangers to human health associated with its consumption. More specifically, the work aims to study the acute and subchronic toxicity of the plant's mixture extract in rats.

## 2. Materials and Methods

### 2.1. Plant Material

The aerial parts of *C. citratus* (leaves and stems) and *B. pilosa* (leaves, stems, and flowers) were collected in Yaoundé (central region of Cameroon), more precisely in Messa-ssi, in February 2018. *C. citratus* was identified by comparison with the botanical collection of D. Dang N°202, registered at the National Herbarium of Yaoundé under N°18626/SFRcam. *B. pilosa* was identified by comparison with the botanical collection of B. Pollar N°2 register at the National Herbarium of Yaoundé under N°60447/HNC.

### 2.2. Preparation of the Aqueous Extract of the Mixture of *B. pilosa* and *C. citratus*

The aerial parts of *B. pilosa* and *C. citratus* were washed, dried in the shade, and then crushed to obtain the powder of each plant. Following the protocol described by Tcheutchoua et al. [17], briefly, 125 g of *B. pilosa* and 200 g of *C. citratus* powder were mixed in 3.25 L of hot distilled water. The mixture was left until cool and then filtered with Whatman paper N°3 and evaporated in an oven (45°C). This made 33 g of aqueous extract form the mixture of *B. pilosa* and *C. citratus* (50 : 50), giving a yield of 10.15%. From the previous study conducted by Tcheutchoua et al. [17], the highest dose of this plant mixture, which was 200 mg/kg was multiplied by 2 and 4 in order to obtain the doses of 400 and 800 mg/kg. These three doses were therefore used in the subchronic study.

### 2.3. Animal Material

Toxicological studies were performed with male and female Wistar albino rats, aged 6–8 weeks and weighing 140–160 g at the start of the experimentation. Females (nulliparous and nonpregnant) and males were separated in cages throughout the studies. The animals were reared in the animal house of the Faculty of Science of the University of Yaoundé I (Cameroun) in Plexiglas cages. The animals were acclimatized for two weeks before the start of the experiment. The animals had free access to tap water as drinking water and to a standard diet (for a 50 kg bag, containing 40% maize, 20% wheat bran, 24% fish meal, 7% palm kernel meal, 4% peanut meal, 2% cottonseed meal, 2% bone meal, 1% table salt, and 20 g vitamin complex). Every effort was made to minimise animal suffering and reduce the number of animals used. The rats were housed in groups of 3 and 5 per cage, respectively, for the acute and subchronic toxicity studies under room temperature (25 ± 3°C), sufficient ventilation, and natural light cycle (12 h/12 h). All procedures and protocols involving animals and their care were conducted in accordance with institutional guidelines and approved by the National Ethics Committee of Cameroon (Reg. No. FWA-IRB00001954).

### 2.4. Evaluation of the Acute Toxicity of the Aqueous Extract of the Mixture of *B. pilosa* and *C. citratus*

The acute toxicity of aqueous extract of the mixture of *B. pilosa* and *C. citratus* was carried out according to the modified OECD guideline 425 [19]. Eighteen rats (9 males and 9 females) were used and divided into 3 batches of 3 females and 3 males each (two test batches and one control batch). The test batches were treated by gavage with the aqueous extract of the mixture of *B. pilosa* and *C. citratus* at doses of 2000 and 5000 mg/kg, respectively, in a single administration. The control lot was given distilled water (10 mL/kg). The animals were observed individually at least once during the first 4 hours. Then, regularly during the first 24 hours and for 14 days after the administration, behaviour (locomotion, aggression, sensitivity to touch, and sound), appearance of faeces and coat condition, and mortality of the animals were observed continuously. The animals were weighed every three days throughout the experimentation period. The weight of the animals obtained was used to calculate the percentage body weight gain change from their initial weights. Weight gain (%) = ((mi-m)/m × 100; *mi* = mass on day *i* (g); and *m* = initial mass (g).

At the end of the experiment, the rats were fasted for 12 hours but had free access to tap water. They were anaesthetised by ether inhalation and sacrificed by cervical dislocation. The rats were then dissected, and organs such as heart, liver, lungs, kidneys, brain, and spleen were well examined, collected, and weighed for the assessment of relative organ weight using the following formula [13]:

(1) $$\begin{array}{c} Wr=\left(wo/wb\right)X\;100\text{,} \end{array}$$

where Wr is the relative organ weight; wo is the organ weight (g); and wb is the rat body weight (g).

### 2.5. Evaluation of the Subchronic Toxicity of the Aqueous Extract of the Mixture of *B. pilosa* and *C. citratus*

This test was performed according to the modified OECD guideline 407 [20]. Six batches of 10 animals each (5 males and 5 females) were used for each dose. Batch 1 received distilled water (10 mL/kg) and batches 2, 3, and 4 received the aqueous extract of the mixture of *B. pilosa* and *C. citratus* (200, 400, and 800 mg/kg), respectively. Animals in batches 5 and 6 (satellite control and satellite batches) were treated by gavage with distilled water (10 mL/kg) and an aqueous extract of the mixture of *B. pilosa* and *C. citratus* at a dose of 800 mg/kg, respectively. Those two batches were observed two more weeks after the other groups. During treatment (28 days), the body weights of the animals were assessed weekly to determine the percentage change in body weight gain, and the signs of toxicity were carefully examined.

### 2.6. Blood and Organ Collection

At the end of the experiment, the rats were made to fast for 12 hours but had free access to tap water. They were then anaesthetised by ether inhalation and sacrificed by decapitation and a part of the arteriovenous blood (around 4 mL) was collected in dry tubes without anticoagulant and allowed to stand for 30 min at room temperature and then centrifuged at 3000 rpm for 15 minutes. The serum obtained was stored in the freezer at −20°C for biochemical analysis. The other part of the arteriovenous blood (around 1 mL) was collected in EDTA (ethylene diamine tetra acetate) tubes for haematological studies. The rats were then dissected, and organs such as the heart, liver, lungs, kidneys, brain, and spleen were collected, rinsed in saline (0.9%), blotted on absorbent paper, and weighed for the assessment of relative organ weights according to the formula previously described in acute toxicity. A portion of these organs was fixed in buffered formalin (4%) for histological analysis.

### 2.7. Biochemical Analysis

Total cholesterol (TC), high-density lipoprotein cholesterol (HDL-Chol), triglyceride (TG) concentrations, uric acid, urea, albumin, and bilirubin levels in serum were determined using commercial diagnostic kits (LABKIT and CHEMELEX S. A. Barcelona) by the colorimetric method of Jaffé [21]. The concentrations of creatinine and activities of alanine and aspartate aminotransferases (ALT and AST) were also determined spectrophotometrically using commercial diagnostic kits (LABKIT and CHEMELEX S. A. Barcelona) by the kinetic method UV-IFCC optimized [21].

### 2.8. Haematological Analysis

The haematological parameters for the blood count were white blood cells (WBC), red blood cells (RBC), lymphocytes (LYM), monocytes (MON), neutrophils (NEUT), eosinophils (EO), basophils (BASO), haemoglobin (HB), haematocrit (HCT), and platelet count (PLT). This analysis was carried out by Medonic Hematology M51 (Beckman Coulter, USA) in the Hematology and Pathology Laboratory of the University Hospital of Yaoundé I (Cameroon) [3].

### 2.9. Histological Analysis

For microscopic evaluation, the organs studied were dehydrated and embedded in paraffin for microscopic examination according to routine laboratory procedures [22]. Paraffin sections of 4 *μ*m were prepared and stained with haematoxylin and eosin (H and E) for histological examination. Morphometric measurements of artery thickness were performed using Image J 1.3 software.

### 2.10. Statistical Analysis

Results were expressed as mean ± SEM. The difference between the groups was compared using a one-way analysis of variance (ANOVA) followed by Turkey's post-test using Graphpad Prism version 8.01. The differences were considered significant from *p*  <  0.05.

## 3. Results

### 3.1. Acute Toxicity

#### 3.1.1. Effects of the Plant Mixture Aqueous Extract on the Behaviour and Mortality of Rats

The effects of the aqueous extract of the mixture of *B. pilosa* and *C. citratus* on the behaviour and mortality of female and male rats are recorded in Table 1. Administration of the aqueous extract of the mixture of *B. pilosa* and *C. citratus* at doses of 2000 and 5000 mg/kg, compared to the control animals, did not induce any apparent signs of toxicity either during the first 4 hours of administration or during the 14 days of experimentation. The extract did not cause any visible changes in coat appearance, aggressiveness, mobility, sensitivity to noise and touch, and the appearance of the faeces and eyes of the test animals as compared to the normal control group. All the animals that started the test were found alive and sound, two weeks after the beginning of the acute toxicity evaluation.

#### 3.1.2. Effects of the Plant Mixture Aqueous Extract on the Relative Organ Weights and Weight Evolution of Rats

**Figure 1:**
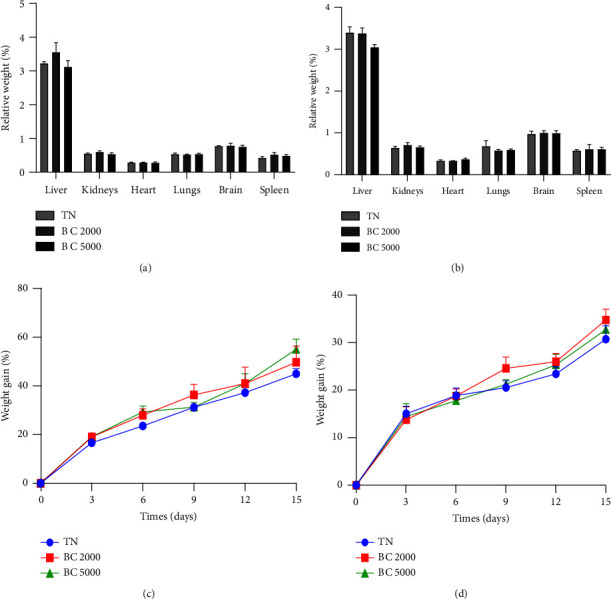
Effects of the plant mixture aqueous extract on the relative organ weight of male (a) and female rats (b) and on the weight gain evolution of male (c) and female (d) rats in acute toxicity. Each bar and point represent the mean ± SEM, *n* = 3; TN = normal rats receiving distilled water (10 mL/kg); BC 2000 and BC 5000 = normal rats treated with the aqueous extract of the mixture of *B. pilosa* and *C. citratus* at doses of 2000 and 5000 mg/kg.

The effects of the aqueous extract of the mixture of *B. pilosa* and *C. citratus* on the relative weight of some organs (A and B) and on weight evolution (C and D) in male and female rats, respectively, are summarised in Figure 1. A single oral dose of the extract at 2000 and 5000 mg/kg in healthy animals of both sexes did not affect significantly the body weight and the relative organ weight of the organs as compared to the normal control.

### 3.2. Subchronic Toxicity

#### 3.2.1. Effects of the Plant Mixture Aqueous Extract on the Body Weight of Male and Female Rats

**Figure 2:**
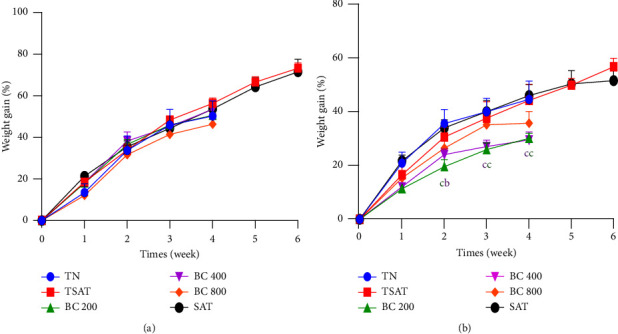
Effects of the plant mixture aqueous extract on the weight evolution of male (a) and female (b) rats in subchronic toxicity. Each point represents the mean ± SEM. *n* = 5. TN: normal rats receiving distilled water (10 mL/kg); TSAT: normal satellite observed 14 more days after stopping every treatment; BC 200, BC 400, and BC 800: animals receiving the aqueous extract of the mixture of *B. pilosa* and *C. citratus* at the respective doses of 200, 400, and 800 mg/kg; SAT: satellite extract at the dose of 800 mg/kg observed 14 days after stopping every treatment. ^b^*p*  <  0.01; ^c^*p*  <  0.001: significant difference from normal controls.

The data recorded in Figures 2(a) and 2(b) summarise the effects of the aqueous extract of the mixture of *B. pilosa* and *C. citratus* on body weight gain in females and males, respectively. The results show that in male rats, no significant differences were observed in all treated batches. In female rats, the weight gain of animals treated with the aqueous extract of the mixture of *B. pilosa* and *C. citratus* at 200 and 400 mg/kg was significantly (*p*  <  0.001) lower than that of the normal control animals from the second week until the end of treatment.

#### 3.2.2. Effects of the Plant Mixture Aqueous Extract on the Relative Weight of Some Organs

Tables 2 and 3 summarise the effects of the aqueous extract of the mixture of *B. pilosa* and *C. citratus* on the relative weight of some organs in female and male rats, respectively. The results show that in female and male rats, no significant difference in the relative weight of all investigated organs was observed in all the treated batches.

#### 3.2.3. Effects of the Plant Mixture Aqueous Extract on Serum Biochemical Parameters

The effects of the aqueous extract of the mixture of *B. pilosa* and *C. citratus* on some serum markers of hepatic and renal functions, respectively, in females and males are summarised in Tables 4 and 5. The aqueous extract at doses of 200 and 400 mg/kg resulted in a significant increase (*p*  <  0.05) in the total cholesterol concentration in females of 21.37% and 21.76%, respectively, compared to normal control (Table 4). For males, Tables 5 shows that chronic administration of the aqueous extract of the mixture of *B. pilosa* and *C. citratus* at doses of 200 and 400 mg/kg resulted in a significant decrease in the concentration of creatinine 13.16% (*p*  <  0.05) and 21.93% (*p*  <  0.001), respectively, compared to normal control animals.

#### 3.2.4. Effects of the Plant Mixture Aqueous Extract on the Elements of the Blood

The effects of the aqueous extract of the mixture of *B. pilosa* and *C. citratus* on some of blood elements are recorded in Tables 6 (female) and 7 (male).  Table 6 shows that administration of the aqueous extract of the mixture of *B. pilosa* and *C. citratus* for 28 days did not affect some blood components. However, the aqueous extract of the mixture of *B. pilosa* and *C. citratus* at a dose of 200 mg/kg induced a significant decrease in white blood cell count (*p*  <  0.05) in females, compared to normal controls. In males, administration of the aqueous extract for 28 days at doses of 200 and 400 mg/kg resulted in a significant (*p*  <  0.05) increase in monocyte count compared to normal controls (Table 7).

#### 3.2.5. Effects of the Plant Mixture Aqueous Extract on the Architecture of the Liver, Kidneys, Spleen, and Lungs

**Figure 3:**
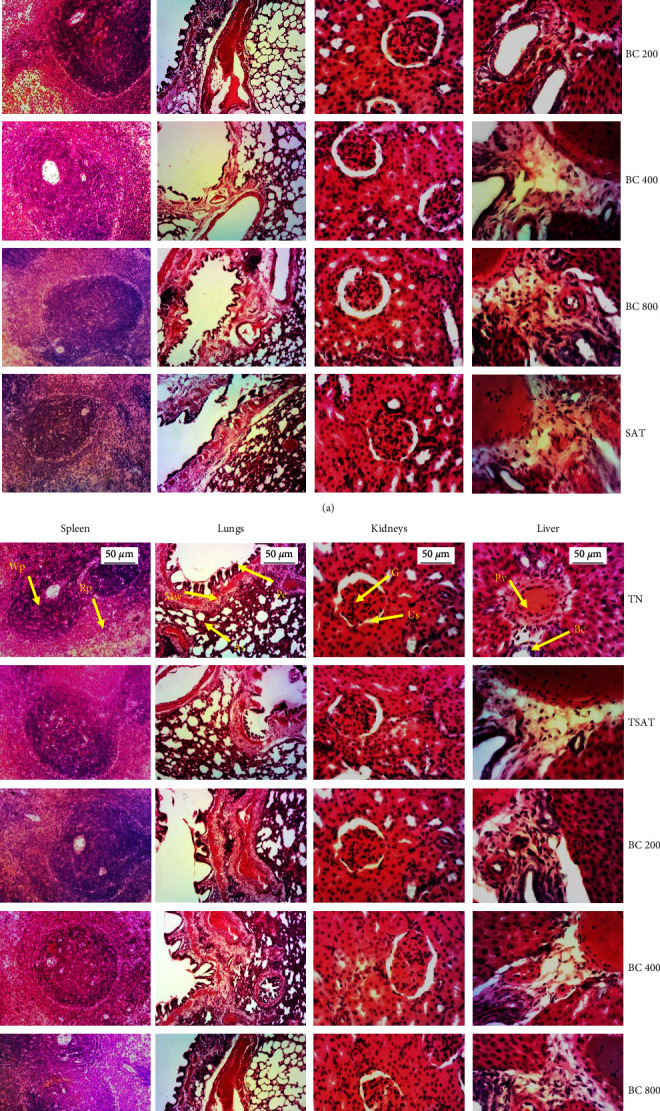
Effects of the plant mixture aqueous extract on the microarchitecture of the liver, kidneys, spleen, and lungs in female (a) and male (b) rats.

Figure 3 shows the effects of the aqueous extract of the mixture of *B. pilosa* and *C. citratus* on the architecture of the liver, kidneys, spleen, and lungs in female (*A*) and male (*B*) rats. Histological sections of the liver of animals in the normal control group and in the groups treated with the aqueous extract of the mixture of *B. pilosa* and *C. citratus* at doses of 200, 400, and 800 mg/kg, as well as those of the satellite groups, showed a normal architecture of the liver parenchyma with a distinct centrilobular vein and hepatocytes. Histology of the kidney showed normal parenchyma with a distinct glomerulus and urinary space. In all treated groups, lung histology showed distinct lung epithelium, alveolar sacs, and muscle wall. Histology of the spleen showed a distinct white and red pulp.

TN: normal rats received distilled water (10 mL/kg); TSAT: normal satellite observed 14 more days after stopping every treatment; BC 200, BC 400, and BC 800: animals receiving the aqueous extract of the mixture of *B. pilosa* and *C. citratus* at the respective doses of 200, 400, and 800 mg/kg; SAT: satellite extract at the dose of 800 mg/kg observed 14 days after stopping every treatment. Liver: Pv = portal vein; He = hepatocyte; Bc = bile canaliculus; kidney: G = glomerulus; Us = urinary space; lungs: Mw = muscle wall; As = alveolar sac; Pe = pulmonary epithelium; spleen: Wp = white pulp; Rp = red pulp. *n* = 5; X : 100X (spleen and lungs), 200X (kidneys and liver).

## 4. Discussion

Studies of toxicity on animals are used to evaluate the potential health risk humans face to unwanted intrinsic effects caused by plant extracts [23]. These secondary effects can appear in the form of biochemical, haematological, histological, or anthropometric alterations [24]. The present study was designed to evaluate the toxicology profile of the aqueous extract of the mixture of *B. pilosa* and *C. citratus* in male and female rats.

One of the problems of phytotherapy is the dose used. Potential toxic effects of natural substances have been reported in several published research works [25, 26]. It is thereby necessary to characterize possible biological effects of every plant used in traditional medicine and their side effects. Therefore, one of our objectives was to verify the toxicity of the aqueous extract of the mixture of *B. Pilosa* and *C. citratus* in acute administration.

The acute toxicity study is the basis for classification and labelling of substances, and the acute toxicity study is the basis for classification and labelling of substances and helps to decide on the dose of compounds to be given in animal studies [26]. In the present work, the acute toxicity focused on the observation of behavioural and physiological changes in rats. The administration of a single dose of the aqueous extract of the mixture of *B. pilosa* and *C. citratus* (2000 and 5000 mg/kg) to rats did not induce any visible signs of toxicity either during the first 4 hours of administration or during the 14 days of experimentation. The fact that all the animals were found alive and sound at the end of the experimental period suggests that the lethal dose 50 (LD_50_) of the plant mixture aqueous extract is above 5000 mg/kg. These findings are in agreement with those of Yinyang Jacques et al. [3] who obtained similar results in the acute toxicity study of the combination of the aqueous extracts of the trunk bark of *Musanga cecropioides* and the fruits of *Picralima nitida*. The abovementioned assessment is not completely true as far the plants are taken alone in the extract. In fact, the acute toxicity of *C. citratus* (2000 mg/kg) was accompanied with symptoms such as torpor, nose, and eyelid bleeding in Wistar rats [6]. Histological examination revealed atrophy of the stomach mucosa and necrosis of the hepatocytes [6], whereas the LD_50_of *C. citratus* was 3250 and 3500 mg/kg in rats and male Swiss mice, respectively [27]. On the other hand, with *B. Pilosa* extract (3500 mg/kg), there were no changes in behaviour, sensory nervous system responses, and gastrointestinal effects in the experimental animals [14]. All the mice survived beyond 24 h of observation; the LD_50_ of the extract was then above 3.5 g/kg [14]. When compared to the extract of isolated plants, the mixture appeared to be more saved. This could be explained with the inhibition of the side effects of *C. citratus* compounds by some compounds found in *B. Pilosa*. According to Hodge and Sterner, the observations in the present study implies that the extract of the combination can be classified as less toxic [28].

Since no toxic effects were observed in the acute study, an additional study was conducted to assess the subchronic toxicity [19] of the aqueous extract of the *B. pilosa* and *C. citratus* mixture during a 28-day experiment on rats. In that second study, the weight loss observed in female rats treated with the aqueous extract of the plant mixture can be explained with the fact that *C. citratus* present in this plant mixture is well known to induce weight loss [10]. Generally, its decoction is advised to pregnant women who are overloaded. The interference with some female hormones could also be incriminated, since no effect on body weight gain was observed in male rats. A sensitive indicator in toxicity studies is the relative organs weight [29]. In the present study, no significant change was observed in the relative organ (heart, liver, spleen, kidney, and lungs) weight of all the rats. These results may then confirm the low toxicity of the aqueous extract of the mixture of *B*. *pilosa* and *C. citratus*.

The liver is involved in the body detoxification, while the kidneys participate in the purification of the blood and the elimination of waste [30]. The assessment of liver and kidney integrity and function is therefore very important in the study of the toxicity of drugs and plant extracts, since they are used for the welfare of the organism [29]. Thus, biochemical analysis carried out in the present study could reveal possible injuries to the liver and kidney induced by the ingestion of the extract. The increase in TC on female rats treated with the aqueous extract of the plant mixture (200 and 400 mg/kg) correlated to the increase in HDL-C can therefore be taken as an improvement for lipid profile. It is true that Hanisa et al. [31] found no significant changes in serum markers of lipid profile in the subchronic toxicity of *C. citratus.* Nonetheless, the observations in the present study are in agreement with our previous study showing that the extract of the mixture improves the lipid profile of hypertensive rats [17]. Transaminases (ALT and AST) are good markers of liver integrity and function [32]. Normally, AST and ALT are enzymes of mitochondrial and cytoplasmic origin. But any cell necrosis, destruction of the hepatic parenchyma or an increase in membrane permeability of the hepatocytes leads to the flow of these enzymes in the blood. That in turn will increase their serum levels [34]. According to Adewale et al. [34], a low level in the liver enzymes (AST and/or ALT) indicates a hepato-protective effect of the plant, which could explain the results observed. In fact, Hanisa et al. [31] observed no significant changes in serum biochemical markers of liver function after 28 days of *C. citratus* infusion administration. The same observations were made in the subchronic toxicity of *B. pilosa* [12]. The results observed in the biochemical markers were confirmed with the histology of the liver. Moreover, renal function can be assessed by testing some biochemical markers such as urea, creatinine, uric acid, and electrolytes [32]. Indeed, these markers have high values in case of renal alteration [25]. The decrease in creatinine levels in male rats having received the aqueous extract of the mixture plants (200 and 400 mg/kg) confirmed therefore the nephroprotective effects already demonstrated in this extract [17]. These results were confirmed by histological analysis which showed no alteration of the renal structure.

Toxicity may manifest itself in the haematopoietic system, in the form of a decrease in the number of circulating cells, functional and structural alterations, and, more rarely, changes in morphology [35]. Therefore, the evaluation of haematological parameters is essential to establish the effects of plant extracts on animal's blood system. Blood parameter analysis is relevant because it provides much information. It can reveal haematopoietic function (myeloid lineage cells), allergies occurrence (white blood cells), or intravascular effects such as haemolysis [36]. The decrease in white blood cells in females and the increase in monocyte count in males having received the aqueous extract of the mixture plant at the dose of 200 mg/kg suggest that the extract did not cause a significant change in haematopoietic function. This could therefore explain why no sign of inflammation was observed in the structure of all investigated organs, suggesting a minor cause of the above difference in blood cells.

## 5. Conclusion

The work in the present study focused on the evaluation of the acute and subchronic toxicity of the aqueous extract of the mixture of *B. pilosa* and *C. citratus*. No sign of toxicity was observed in acute toxicity study with the oral administration of the dose 5000 mg/kg or less. No morphological and behavioural signs of toxicity were observed for both acute and subchronic toxicity. In subchronic toxicity, the extract stimulated weight growth in male. The extract did not induce any change in relative organ weights at all doses tested. Furthermore, it did not induce any significant changes in liver and kidney function; nonetheless, it improved the lipid profile. This study supports the production of an improved traditional medicine after preclinical testing.

## Figures and Tables

**Table 1:** Effects of the plant mixture aqueous extract on behaviour and mortality rate of rats during the acute toxicity study.

Groups			Locomotion	Sensitivity			Stool appearance	Coat condition	Sample size	
				To touch	Noise	Aggressiveness			Initial number	Final number
TN 10 mL/kg	Females	1 h	*N*	*N*	*N*	*N*	*G*	*N*	3	3
		4 h	*N*	*N*	*N*	*N*	*G*	*N*	3	3
		24 h	*N*	*N*	*N*	*N*	*G*	*N*	3	3
		14 days	*N*	*N*	*N*	*N*	*G*	*N*	3	3
	Males	1 h	*N*	*N*	*N*	*N*	*G*	*N*	3	3
		4 h	*N*	*N*	*N*	*N*	*G*	*N*	3	3
		24 h	*N*	*N*	*N*	*N*	*G*	*N*	3	3
		14 days	*N*	*N*	*N*	*N*	*G*	*N*	3	3

BC 2000 mg/kg	Females	1 h	*N*	*N*	*N*	*N*	*G*	*N*	3	3
		4 h	*N*	*N*	*N*	*N*	*G*	*N*	3	3
		24 h	*N*	*N*	*N*	*N*	*G*	*N*	3	3
		14 days	*N*	*N*	*N*	*N*	*G*	*N*	3	3
	Males	1 h	*N*	*N*	*N*	*N*	*G*	*N*	3	3
		4 h	*N*	*N*	*N*	*N*	*G*	*N*	3	3
		24 h	*N*	*N*	*N*	*N*	*G*	*N*	3	3
		14 days	*N*	*N*	*N*	*N*	*G*	*N*	3	3

BC 5000 mg/kg	Females	1 h	*N*	*N*	*N*	*N*	*G*	*N*	3	3
		4 h	*N*	*N*	*N*	*N*	*G*	*N*	3	3
		24 h	*N*	*N*	*N*	*N*	*G*	*N*	3	3
		14 days	*N*	*N*	*N*	*N*	*G*	*N*	3	3
	Males	1 h	*N*	*N*	*N*	*N*	*G*	*N*	3	3
		4 h	*N*	*N*	*N*	*N*	*G*	*N*	3	3
		24 h	*N*	*N*	*N*	*N*	*G*	*N*	3	3
		14 days	*N*	*N*	*N*	*N*	*G*	*N*	3	3

Reaction: normal (*N*); stool appearance: granular (*G*). TN = normal rats receiving distilled water (10 mL/kg); BC 2000 and BC 5000 = normal rats treated with the aqueous extract of the mixture of *B. pilosa* and *C. citratus* at doses of 2000 and 5000 mg/kg.

**Table 2:** Effects of the plant mixture aqueous extract on the relative weight of organs of females.

Organs		TN	TSAT	BC 200	BC 400	BC 800	SAT
Relative weight (%)	Liver	2.92 ± 0.05	2.92 ± 0.06	2.90 ± 0.06	2.82 ± 0.07	2.92 ± 0.11	3.04 ± 0.16
	Kidneys	0.59 ± 0.02	0.61 ± 0.04	0.57 ± 0.02	0.61 ± 0.03	0.64 ± 0.02	0.65 ± 0.04
	Heart	0.31 ± 0.01	0.30 ± 0.01	0.32 ± 0.01	0.29 ± 0.01	0.30 ± 0.01	0.31 ± 0.02
	Lungs	0.63 ± 0.05	0.58 ± 0.06	0.65 ± 0.03	0.66 ± 0.06	0.60 ± 0.07	0.67 ± 0.06
	Brain	0.85 ± 0.03	0.81 ± 0.02	0.85 ± 0.01	0.85 ± 0.02	0.83 ± 0.02	0.85 ± 0.02
	Spleen	0.43 ± 0.04	0.44 ± 0.01	0.41 ± 0.03	0.44 ± 0.03	0.47 ± 0.07	0.49 ± 0.03

Each value represents the mean ± SEM, *n* = 5. TN: normal rats receiving distilled water (10 mL/kg); TSAT: normal satellite observed 14 more days after stopping every treatment; BC 200, BC 400, and BC 800: animals receiving the aqueous extract of the mixture of *B. pilosa* and *C. citratus* at the respective doses of 200, 400, and 800 mg/kg; SAT: satellite extract at the dose of 800 mg/kg observed 14 days after stopping every treatment.

**Table 3:** Effects of the plant mixture aqueous extract on the relative weight of the organs of males.

Organs		TN	TSAT	BC 200	BC 400	BC 800	SAT
Relative weight (%)	Liver	3.01 ± 0.11	2.87 ± 0.08	2.70 ± 0.16	2.83 ± 0.12	2.69 ± 0.06	2.64 ± 0.08
	Kidneys	0.53 ± 0.03	0.50 ± 0.01	0.57 ± 0.03	0.55 ± 0.01	0.54 ± 0.03	0.51 ± 0.02
	Heart	0.29 ± 0.01	0.29 ± 0.02	0.30 ± 0.01	0.31 ± 0.01	0.30 ± 0.02	0.28 ± 0.02
	Lungs	0.63 ± 0.09	0.54 ± 0.04	0.63 ± 0.05	0.60 ± 0.06	0.59 ± 0.05	0.58 ± 0.05
	Brain	0.66 ± 0.04	0.65 ± 0.01	0.71 ± 0.02	0.71 ± 0.02	0.72 ± 0.01	0.68 ± 0.03
	Spleen	0.42 ± 0.02	0.32 ± 0.01	0.35 ± 0.02	0.37 ± 0.01	0.38 ± 0.05	0.36 ± 0.02

Each value represents the mean ± SEM. *n* = 5. TN: normal rats receiving distilled water (10 mL/kg); TSAT: normal satellite observed 14 more days after stopping every treatment; BC 200, BC 400, and BC 800: animals receiving the aqueous extract of the mixture of *B. pilosa* and *C. citratus* at the respective doses of 200, 400, and 800 mg/kg; SAT: satellite extract at the dose of 800 mg/kg observed 14 days after stopping every treatment.

**Table 4:** Effects of the plant mixture aqueous extract on serum biochemical parameters in females.

Parameters	TN	TSAT	BC 200	BC 400	BC 800	SAT
TP (mg/dL)	0.90 ± 0.51	0.95 ± 0.46	0.88 ± 0.14	0.93 ± 0.57	0.93 ± 0.24	0.98 ± 0.19
AST (U/L)	71.80 ± 5.79	76.70 ± 6.34	74.50 ± 5.77	79.60 ± 5.41	80.40 ± 5.76	75.80 ± 4.69
ALT (U/L)	34.29 ± 2.62	31.06 ± 1.47	33.93 ± 2.52	40.49 ± 3.74	41.57 ± 2.48	39.3 ± 4.92
TB (mg/dL)	0.37 ± 0.09	0.66 ± 0.32	0.55 ± 0.20	0.90 ± 0.18	0.66 ± 0.29	0.46 ± 0.16
Creatinine (mg/dL)	0.99 ± 0.04	0.98 ± 0.04	0.94 ± 0.03	0.90 ± 0.03	0.92 ± 0.03	0.90 ± 0.02
Urea (mg/dL)	33.51 ± 1.04	35.58 ± 0.44	32.45 ± 0.23	30.08 ± 0.63	37.92 ± 0.21	36.20 ± 0.32
Uric acid (mg/dL)	2.89 ± 0.14	3.08 ± 0.09	2.94 ± 0.13	3.00 ± 0.33	2.92 ± 0.12	2.80 ± 0.07
TC (mg/dL)	54.07 ± 2.76	49.79 ± 5.38	65.63 ± 4.81^a^	65.84 ± 3.62^a^	43.82 ± 2.87	42.34 ± 4.69
TG (mg/dL)	36.13 ± 1.34	38.93 ± 1.58	40.08 ± 3.21	43.88 ± 4.30	36.91 ± 2.18	34.07 ± 1.90
HDL-C (mg/dL)	34.45 ± 1.68	27.89 ± 0.85	34.90 ± 2.80	42.37 ± 4.79	35.44 ± 2.18	30.21 ± 2.90

Each value represents the mean ± SEM, *n* = 5. TN: normal rats receiving distilled water (10 mL/kg); TSAT: normal satellite observed 14 more days after stopping every treatment; BC 200, BC 400, and BC 800: animals receiving the aqueous extract of the mixture of *B. pilosa* and *C. citratus* at the respective doses of 200, 400, and 800 mg/kg; SAT: satellite extract at the dose of 800 mg/kg observed 14 days after stopping every treatment. TP: total protein; ALT: alanine aminotransferase; AST: aspartate aminotransferase. TB: total bilirubin; TC: total cholesterol; HDL-C: high-density lipoprotein cholesterol; TG: triglycerides. ^a^*p*  <  0.05: significant difference from normal controls.

**Table 5:** Effects of the plant mixture aqueous extract on serum biochemical parameters in males.

Parameters	TN	TSAT	BC 200	BC 400	BC 800	SAT
TP (mg/dL)	0.92 ± 0.51	0.97 ± 0.29	0.82 ± 0.15	0.93 ± 0.71	0.96 ± 0.29	0.99 ± 0.38
AST (U/L)	70.20 ± 2.62	79.60 ± 3.57	67.00 ± 4.52	71.00 ± 4.98	69.30 ± 4.27	72.10 ± 3.59
ALT (U/L)	32.94 ± 2.16	35.96 ± 4.97	31.12 ± 1.70	32.34 ± 2.34	35.49 ± 5.39	31.31 ± 2.29
TB (mg/dL)	1.57 ± 0.33	1.24 ± 0.40	1.16 ± 0.26	1.23 ± 0.12	0.90 ± 0.10	1.80 ± 0.30
Creatinine (mg/dL)	1.14 ± 0.03	1.08 ± 0.01	0.99 ± 0.03^a^	0.89 ± 0.04^c^	1.01 ± 0.01	1.11 ± 0.03
Urea (mg/dL)	30.89 ± 0.53	31.67 ± 0.43	33.50 ± 0.20	33.09 ± 0.34	33.52 ± 0.21	30.24 ± 0.56
Uric acid (mg/dL)	3.89 ± 0.37	3.58 ± 0.45	3.54 ± 0.23	3.60 ± 0.80	3.92 ± 0.40	2.98 ± 0.27
TC (mg/dL)	72.83 ± 2.49	80.07 ± 3.47	72.24 ± 3.97	74.34 ± 2.38	77.14 ± 3.36	72.21 ± 3.92
TG (mg/dL)	41.55 ± 2.07	43.36 ± 2.10	34.31 ± 3.57	41.49 ± 2.37	33.51 ± 3.86	42.64 ± 3.09
HDL-C (mg/dL)	39.45 ± 2.65	46.18 ± 2.78	37.16 ± 2.96	34.99 ± 3.12	33.91 ± 2.28	31.74 ± 2.20

Each value represents the mean ± SEM. *n* = 5. TN: normal rats receiving distilled water (10 mL/kg); TSAT: normal satellite observed 14 more days after stopping every treatment; BC 200, BC 400, and BC 800: animals receiving the aqueous extract of the mixture of *B. pilosa* and *C. citratus* at the respective doses of 200, 400, and 800 mg/kg; SAT: satellite extract at the dose of 800 mg/kg observed 14 days after stopping every treatment. TP: total protein; ALT: alanine aminotransferase; AST: aspartate aminotransferase. TB: total bilirubin; TC: total cholesterol; HDL-C: high-density lipoprotein cholesterol; TG: triglycerides. ^a^*p*  <  0.05, ^c^*p*  <  0.001: significant difference from normal controls.

**Table 6:** Effects of the plant mixture aqueous extract on the blood elements in females.

Parameters	TN	TSAT	BC 200	BC 400	BC 800	SAT
WBC (*∗*10^3^/*μ*L)	14.65 ± 1.50	13.36 ± 0.68	9.68 ± 0.79^a^	13.70 ± 0.87	13.81 ± 0.34	14.84 ± 0.34
RBC (*∗*10^3^/*μ*L)	8.43 ± 0.11	8.01 ± 0.21	8.29 ± 0.21	8.45 ± 0.20	7.95 ± 0.23	8.18 ± 0.47
LYM (*∗*10^3^/*μ*L)	11.46 ± 0.94	9.60 ± 0.81	8.47 ± 1.45	5.23 ± 2.06	8.67 ± 0.76	7.44 ± 1.51
MONO (*∗*10^3^/*μ*L)	0.36 ± 0.04	0.37 ± 0.01	0.31 ± 0.03	0.35 ± 0.05	0.32 ± 0.09	0.29 ± 0.12
NEUT (*∗*10^3^/*μ*L)	3.87 ± 0.75	2.93 ± 0.13	2.84 ± 0.80	4.02 ± 0.83	3.31 ± 0.60	3.65 ± 0.25
EO (*∗*10 ^3^/*μ*L)	0.24 ± 0.06	0.26 ± 0.07	0.24 ± 0.03	0.12 ± 0.02	0.22 ± 0.12	0.17 ± 0.07
BASO (*∗*10^3^/*μ*L)	0.02 ± 0.01	0.02 ± 0.01	0.01 ± 0.01	0.01 ± 0.01	0.01 ± 0.01	0.01 ± 0.01
HB (g/dL)	14.27 ± 0.27	13.50 ± 0.46	13.90 ± 0.30	14.33 ± 0.43	13.73 ± 0.15	13.43 ± 0.67
PLT (*∗*10^3^/*μ*L)	853.50 ± 77.50	755.50 ± 59.50	871.00 ± 40.00	951.30 ± 67.98	926.70 ± 30.47	1003 ± 11.50
HCT (%)	51.57 ± 0.80	49.27 ± 1.18	50.50 ± 1.61	52.37 ± 1.02	51.20 ± 1.45	49.13 ± 2.96

Each value represents the mean ± SEM. *n* = 5. TN: normal rats receiving distilled water (10 mL/kg); TSAT: normal satellite observed 14 more days after stopping every treatment; BC 200, BC 400, and BC 800: animals receiving the aqueous extract of the mixture of *B. pilosa* and *C. citratus* at the respective doses of 200, 400, and 800 mg/kg; SAT: satellite extract at the dose of 800 mg/kg observed 14 days after stopping every treatment. WBC: white blood cells; RBC: red blood cells; LYM: lymphocytes; MON: monocytes; NEUT: neutrophils; EO: eosinophils; BASO: basophils; PLT: blood platelets; HB: haemoglobin; HCT: haematocrit. ^a^*p*  <  0.05: significant difference from normal controls.

**Table 7:** Effects of the plant mixture aqueous extract on the blood elements in males.

Parameters	TN	TSAT	BC 200	BC 400	BC 800	SAT
WBC (*∗*10^3^/*μ*L)	12.23 ± 0.87	10.97 ± 0.38	13.05 ± 0.55	15.58 ± 0.85	18.32 ± 1.36^a^	13.70 ± 0.94
RBC (*∗*10^3^/*μ*L)	8.77 ± 0.21	8.75 ± 0.24	8.39 ± 0.42	8.98 ± 0.07	8.57 ± 0.48	9.57 ± 0.17
LYM (*∗*10^3^/*μ*L)	8.55 ± 0.67	7.01 ± 0.09	8.86 ± 0.69	10.99 ± 0.50	12.65 ± 1.04	12.16 ± 1.11
MONO (*∗*10^3^/*μ*L)	0.29 ± 0.07	0.40 ± 0.22	0.82 ± 0.15^a^	0.61 ± 0.09^a^	0.39 ± 0.04	0.35 ± 0.01
NEUT (*∗*10^3^/*μ*L)	3.29 ± 0.21	3.84 ± 0.67	3.36 ± 0.84	4.22 ± 0.47	4.35 ± 0.68	3.50 ± 0.25
EO (*∗*10^3^/*μ*L)	0.09 ± 0.01	0.22 ± 0.08	0.04 ± 0.02	0.14 ± 0.04	0.17 ± 0.02	0.22 ± 0.09
BASO (*∗*10^3^/*μ*L)	0.02 ± 0.01	0.02 ± 0.01	0.01 ± 0.01	0.01 ± 0.01	0.01 ± 0.01	0.01 ± 0.01
HB (g/dL)	15.10 ± 0.11	14.60 ± 0.69	14.60 ± 0.12	14.97 ± 0.28	14.47 ± 0.24	15.73 ± 0.32
PLT (*∗*10^3^/*μ*L)	816.00 ± 87.00	920.70 ± 46.24	904.30 ± 20.41	869.70 ± 35.35	1023.00 ± 20.50	759.00 ± 83.00
HCT (%)	55.07 ± 1.06	53.30 ± 2.94	55.23 ± 1.19	55.87 ± 1.14	54.53 ± 0.83	57.10 ± 1.22

Each value represents the mean ± SEM. *n* = 5. TN: normal rats receiving distilled water (10 mL/kg); TSAT: normal satellite observed 14 more days after stopping every treatment; BC 200, BC 400, and BC 800: animals receiving the aqueous extract of the mixture of *B. pilosa* and *C. citratus* at the respective doses of 200, 400, and 800 mg/kg; SAT: satellite extract at the dose of 800 mg/kg observed 14 days after stopping every treatment. WBC: white blood cells; RBC: red blood cells; LYM: lymphocytes; MON: monocytes; NEUT: neutrophils; EO: eosinophils; BASO: basophils; PLT: blood platelets; HB: haemoglobin; HCT: haematocrit. ^a^*p*  <  0.05: significant difference from normal controls.

## Data Availability

The data used to support the findings of this study are available from the corresponding author upon request.
